# Lichen and Its Microbiome as an Untapped Source of Anti‐Biofilm Compounds

**DOI:** 10.1002/cbdv.202401557

**Published:** 2024-12-20

**Authors:** Marion Millot, Christine Imbert, Christelle Pouget, Marion Girardot, Lengo Mambu

**Affiliations:** ^1^ Laboratoire des Agroressources, Biomolécules et Chimie pour l'Innovation en Santé (LABCiS), UR 22722 Université de Limoges Limoges France; ^2^ Laboratoire Ecologie et Biologie des Interactions (EBI) UMR CNRS 7267, Université de Poitiers Poitiers France

**Keywords:** antimicrobial, associated fungi, bacterial biofilms, fungal biofilms, lichens

## Abstract

Lichen substances have been first described in the 1870s, and around 10 000 compounds have been isolated and characterized. Most of them have been evaluated for their activity on planktonic microorganisms (bacteria and fungi). More recently, microorganisms colonizing the lichen thallus have been isolated and identified using DNA sequencing, giving access to a wide diversity of culturable microorganisms. The increasing research in lichen‐associated microbiomes in recent years has emphasized a wide range of metabolites as a potential source of bioactive compounds. In parallel, humans are facing microbial resistance to conventional antimicrobial drugs. One of the reasons is the biofilm lifestyle of microorganisms. Indeed, the aggregation of microbial communities inside biofilms is now well known and characterized, and some possible ways to fight and destroy biofilms are identified (quorum sensing inhibitors, etc.). The present review aims to summarize the anti‐biofilm potential of lichen metabolites and those from their associated microorganisms (bacteria and/or fungi). Are the metabolites isolated from lichens and their associated fungi displaying any anti‐biofilm activity? This literature synthesis highlights the metabolites of interest as new anti‐biofilm drugs and shows the lack of current biological research dealing with biofilm and lichen metabolites. Acetone and ethyl acetate extracts are the most studied sources of anti‐biofilm agents. Only two lichen metabolites, usnic acid and evernic acid, have been evaluated both as antifungal and antibacterial biofilm compounds. Terpenoids from lichens are still poorly explored for this activity.

## Graphical abstract

Lichens and their associated fungi give rise to the evaluation of several extracts and compounds against biofilms.

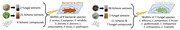

## 1 Introduction

In the fungal kingdom, lichens are unique organisms that are responsible for the production of singular secondary metabolites [1]. This unique life form, which is a symbiosis between fungi (mycobiont) and algae and/or cyanobacteria (photobionts), is considered to be the earliest colonizer of terrestrial habitats on earth [2]. Nowadays, 25 000 different species of lichens inhabit over 8% of the earth's surface, from arctic to tropical regions and from the plains to the highest mountains [3, 4]. The specific, even extreme, conditions of their existence (slow growth and long duration) are consistent with the production of defense metabolites that fight against different biotic and abiotic factors. However, lichens are more than just a single organism; they make up a complex holobiont involving several additional bacterial and fungal communities. Over recent decades, the communities resulting from this mutualist symbiosis have been increasingly studied by scientists (from less than 5 articles per year before 2012 to more than 10 per year in 2022 based on a search of the PubMed database). Thanks to this, the lichen‐associated microbiome (endo‐ and epilichenic fungi, yeasts, and bacteria) is being progressively identified and described. The fungal classes, Dothideomycetes, Eurotiomycetes, Leotiomycetes, Sordariomycetes, and Tremellomycetes, predominate the fungal communities in lichens [5, 6]. Furthermore, distinct communities have been observed between the epilichenic fungi (living in the upper thallus surface) and the endolichenic fungi (living inside the lichen thallus) [7]. According to literature data, lichen bacterial communities are dominated by *Alphaproteobacteria* [8]. The interactions developed among these communities lead to the production of a wide diversity of metabolites endowed with a variety of biological effects [9, 10, 11]. The antimicrobial effects of lichenic extracts and compounds have been extensively evaluated, whereas those of the lichen‐associated microbiome are just beginning to be studied and are revealing some interesting metabolites [12].

While researching new antimicrobial drugs, and faced with microbial resistance, scientists have recently pointed out the importance of considering the biofilm‐living form of microorganisms. Indeed, in addition to the well‐known efflux pump issue, resistance and tolerance strategies developed in biofilms result in low penetration of the antimicrobial drugs [13, 14]. On the one hand, drugs are sequestrated by the exopolysaccharides matrix of the biofilm, and on the other hand, the metabolic activity of certain microorganisms, especially those in the deeper layers of the biofilm, is very low. These mechanisms are the consequences of the multicellular nature of biofilms, which leads to the antimicrobial drug resistance and tolerance of biofilm communities [15].

The National Institute of Health (NIH) revealed that among all acute and chronic infections, 65% and 80%, respectively, are associated with biofilm formation [16]. Staphylococci and enterococci are responsible for frequent cases of hospital‐acquired infections involving a biofilm. Coagulase‐negative staphylococci (*Staphylococcus epidermidis*, *Staphylococcus lugdunensis*, and *Staphylococcus haemolyticus*) and *Staphylococcus aureus* are the predominant bacterial species connected to device‐associated infections involving biofilms. Enterococci have also emerged in recent years as pathogens associated with serious nosocomial infections and biofilms [17]. Generally, bacterial biofilms are associated with resistance against the host's immune system and antibiotics [18].

Fungi also play a significant role in the epidemiology of biofilms and biofilm‐related infections, and in the lack of efficacy of antifungal agents in treating certain patients [19, 20]: yeasts and/or filamentous fungi can be isolated from biofilms associated with many medical devices and cellular surfaces. Yeasts belonging to the *Candida* genus are the most studied in this context, in particular, *Candida albicans*, but biofilms formed by fungi of the *Aspergillus*, *Cryptococcus*, and other genera of medical interest are also studied. *C. albicans* belongs to human microflora and is a major opportunistic human pathogen [21]. This species is, for example, responsible for candidemia, which is often reported in patients with vascular catheters, and the presence of these medical devices is one of the main risk factors for the development of a biofilm and, consequently, a biofilm‐related infection. In fact, in real life conditions, most biofilms are polymicrobial, mixing bacteria and fungi, or different bacterial species, or, more rarely, different fungal species [22]. The polymicrobial nature of biofilms is able to significantly influence the efficacy of antimicrobial molecules [23], especially due to communication and interaction implemented by microorganisms in these complex microbial communities.

**DIAGRAM 1:**
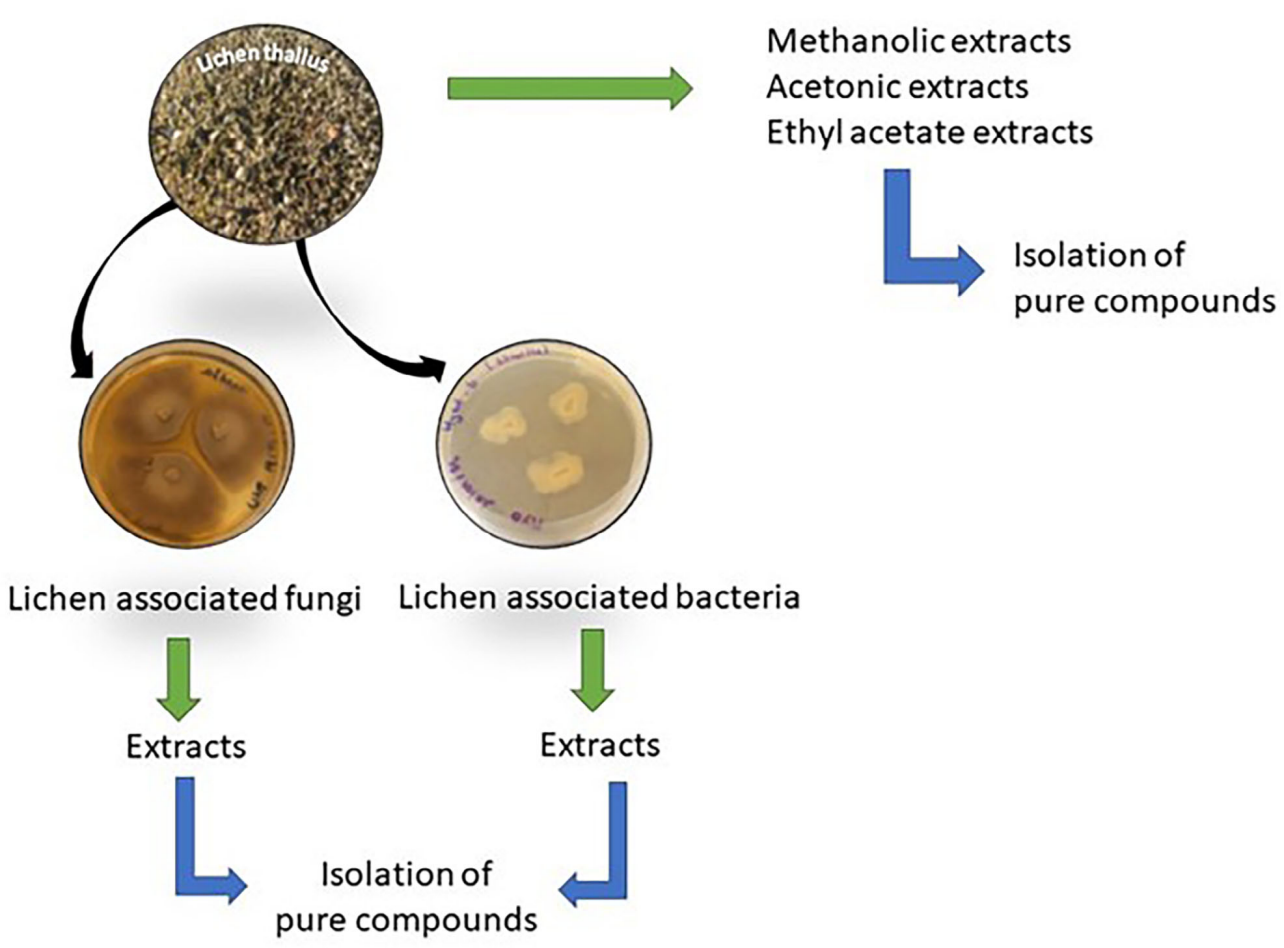
Obtaining natural products from lichens and their associated microorganisms.

A parallel can be drawn between lichen and biofilm, and similarities can be listed: an adherence stage to a hydrophobic surface; interactions between microbial communities; the existence of an extracellular matrix (a mixture of exopolysaccharides, proteins, and nucleic acids for biofilm and a mucilaginous matrix for lichen thallus); and the role of community growth in resistance [24]. Therefore, the study of biofilms and of strategies to eliminate them through the discovery of novel bioactive metabolites is a major research field. Many reviews on anti‐biofilm compounds already exist. However, for the first time, this review aims to point out the anti‐biofilm activities of extracts or compounds obtained from lichens or their related fungi or bacteria (Diagram 1).

**FIGURE 1:**
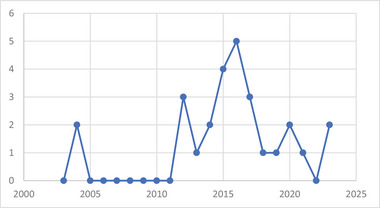
Number of articles associating “lichen,” “lichen endophytes,” endolichenic, or “lichen compounds” with “biofilms.”

Although research on lichen chemistry has been carried out since the end of the 19th century, the knowledge and studies on endophytic microorganisms as well as the biofilm‐living form are more recent. These aspects resulted in few publications over a limited period (2003–2023) (Figure 1).

It has been found that, more often than not, the anti‐biofilm efficacy of antimicrobial agents is assessed by determining the minimum biofilm inhibitory concentration (MBIC) and the minimum biofilm eradicating concentration (MBEC) in vitro, in microplates [25], in single‐species conditions and, more rarely, in polymicrobial ones. Unfortunately, this quantitative assessment of the anti‐biofilm activity cannot be performed in routine conditions for diagnostic purposes, as it would be too costly in terms of both time and materials. However, it is well known that for a given fungal isolate, the MBIC and MBEC values obtained are usually far higher than the minimum inhibitory concentration (MIC) values and minimum bactericidal concentration or minimum fungicidal concentration, which has been traditionally assessed in routine conditions. This gap demonstrates the different behavior adopted by microorganisms depending on whether they grow in a planktonic or sessile (biofilm) mode.

Furthermore, tests for MBIC and MBEC are affected by a lack of standardization. Indeed, even if the protocols described are broadly similar, experimental conditions may vary from one research laboratory to another: the duration of the adhesion phase, the culture media, the substrate, and so on [26].

## 2 Extracts and Compounds With Activity Against Bacterial Biofilms

### 2.1 Extracts

#### 2.1.1 Lichenic Extracts

Lichen extracts have been widely studied for their antimicrobial properties, but their anti‐biofilm potential is still poorly explored. The few articles describing these activities are presented in the paragraphs below and summarized in Table 1a. The anti‐biofilm potential of acetone, ethyl acetate, and methanol extracts of two *Parmeliaceae* species—the lichens *Platismatia glauca* and *Pseudevernia furfuracea*—was evaluated by Mitrovic et al. using peg lid and against a 20‐h‐old biofilm [10, 27]. The acetone and ethyl acetate extracts of *P. glauca* showed the best anti‐biofilm activities on *S. aureus* and *Proteus mirabilis* with an MBIC value of 630 µg/mL. On the other hand, the methanol extract of *P. furfuracea* was efficient with MBIC at 1250 µg/mL on *S. aureus* and 630 µg/mL on *P. mirabilis*. Isolated compounds were not individually tested, but GC–MS analyses revealed caperatic acid, atraric acid, atranorin, and chloroatranorin as the predominant compounds in *P. glauca*, whereas atraric acid, olivetoric acid, atranorin, and chloroatranorin were the major constituents in the *P. furfuracea* extract [27]. The same researchers explored the biological activities of two frequent lichen species in South East Serbia—*Cladonia foliacea* and *Hypogymnia physodes*—on the same bacterial biofilms (*S. aureus* and *P. mirabilis*) with the same methodology [28]. The anti‐biofilm activity was confirmed for both lichens with more prominent results on the Gram‐positive bacteria *S. aureus* (MBIC values between 80 and 1600 µg/mL) than on *P. mirabilis* (MBIC values ranging between 1250 and 2500 µg/mL). Methanol and ethyl acetate extracts of *C. foliacea* showed the best activities with MBEC values at 80 µg/mL against *S. aureus*. Chemical profiling using GC and GC/MS allowed for the identification of the major compounds of each lichenic extract. Methanol and ethyl acetate extracts of *H. physodes* contained mostly atraric acid, olivetol, and atranol, whereas usnic acid was the major component in all *C. foliacea* extracts. These compounds could be involved in the observed anti‐biofilm activity. In a comparative survey, Özyiğitoğlu et al. investigated the antibacterial and anti‐biofilm activities of *Hypogymnia tubulosa* extracts, collected in different areas of Turkey [29]. Results showed that chloroform–methanol–acetone (CMA 1:1:1) extract provided significant anti‐maturation activity against two pathogenic microbial species—*S. aureus* and *Enterococcus faecalis*—at varying levels depending on the lichen locality. Even if the chemical content of the extracts has not been analyzed, the authors supposed that this anti‐biofilm activity of CMA extract may be due to the presence of atranorin or usnic acid as suggested by Pompilio et al. and Cansaran‐Duman et al. [30, 31]. In general, they concluded that extracts with low antibacterial activity showed a higher inhibition effect on biofilm. Oakmoss absolutes obtained from the lichen *Evernia prunastri* were investigated on *Legionella pneumophila*. Kondo et al. investigated activity against both planktonic and sessile bacteria. First, they revealed their effect against planktonic bacteria (minimal inhibitory concentration [MIC] ranging between 7.3 and 16 µg/mL) [32]. Then, they investigated their effect on *L. pneumophila* biofilm formation and assessed their bactericidal activity against the biofilm cells. At a concentration of 0.5 × MIC, the two tested absolutes enhanced the biofilm formation by 136.8% ± 18.9% and 132.9% ± 15.9%, respectively, compared to a positive control, and showed no bactericidal activity against sessile cells (≥256.0 µg/mL after 48 h of treatment) [33]. These extracts therefore had a biofilm‐promoting effect rather than a destructive one. Five *Cetrelia* species (*Cetrelia japonica*, *Cetrelia olivetorum*, *Cetrelia braunsiana*, *Cetrelia chicitae*, and *Cetrelia delavayana*) were cultured on malt–yeast agar plates to obtain ethyl acetate extracts [34]. These extracts were evaluated for their effect on *Pseudomonas aeruginosa* quorum sensing (QS). All extracts have shown an inhibition of QS, but the highest inhibition was observed for *C. braunsiana* ethyl acetate extract (63.8% of QS inhibition at 24.7 µg/mL). No effect was observed against planktonic bacteria. More recently, a research group made attempts to explore the antimicrobial and anti‐biofilm properties of different types of lichen‐based nanoparticles (NPs). Alavi et al. observed the anti‐biofilm activity of *Protoparmeliopsis muralis* aqueous extract NPs (Ag, Cu, TiO_2_, ZnO, and Fe_3_O_4_). For Ag, Cu, and ZnO NPs, the reduction of the biofilm biomass for a concentration of 100 µg/mL was higher. Ag NPs were more effective against *P. aeruginosa* biofilms [35].

**TABLE 1a:** Lichen and lichenic microbiome extracts and their activity against bacterial biofilms.

Targeted bacterial strain	Targeted biofilm phase	Source of extracts	Type of extract	Quantification of the activity on biofilm	Mechanism of action	Refs.
	**Lichens**					
*Staphylococcus aureus* ATCC 25923	Mature biofilm (20‐h‐old biofilm)	*Platismatia glauca*	Acetone	MBIC = MBEC 0.63 mg/mL	ND	[3]
			Ethyl acetate	MBIC 0.63 mg/mL MBEC 1.25 mg/mL		
			Methanol	MBIC = MBEC 2.5 mg/mL		
		*Cladonia foliacea*	Acetone	MBIC 0.63 mg/mL MBEC 0.08 mg/mL		[28]
			Ethyl acetate	MBIC 1.25 mg/mL MBEC 0.08 mg/mL		
			Methanol	MBIC 2.5 mg/mL MBEC 0.16 mg/mL		
		*Hypogymnia physodes*	Acetone	MBIC 2.5 mg/mL MBEC 0.31 mg/mL		
			Ethyl acetate	MBIC = MBEC 0.31 mg/mL		
			Methanol	MBIC = MBEC = 0.31 mg/mL		
*Proteus mirabilis* ATCC 12453	Mature biofilm (20‐h‐old biofilm)	*Pseudevernia furfuracea*	Acetone	BIC 1.25 mg/mL BIC 0.63 mg/mL	ND	[10]
			Ethyl acetate	MBIC 2.5 mg/mL MBEC 0.31 mg/mL		
			Methanol	MBIC 1.25 mg/mL MBEC 0.31 mg/mL		
		*Cladonia foliacea*	Acetone	MBIC 2.5 mg/mL MBEC > 2.5 mg/mL		[28]
			Ethyl acetate	MBIC 1.25 mg/mL MBEC > 2.5 mg/mL		
			Methanol	MBIC 2.5 mg/mL MBEC > 2.5 mg/mL		
		*Hypogymnia physodes*	Acetone	MBIC = MBEC = 2.5 mg/mL		
*Legionella pneumophila* Philadelphia 1 strain JCM7571	Biofilm formation	*Evernia prunastri*	Ethyl acetate	MBIC = MBEC = 2.5 mg/mL	ND	[33]
*Staphylococcus aureus* ATCC 25923	Biofilm formation	*Hypogymnia tubulosa*	Chloroform/Methanol/Acetone (1:1:1)	OD measurement: OD: 0.036–0.351	ND	[29]
*Enterococcus faecalis* ATCC 29212	Biofilm formation	*Hypogymnia tubulosa*	Chloroform/Methanol/Acetone (1:1:1)	OD measurement: OD: 0.038–0.091	ND	[29]
	**Cultured mycobiont**					
*Pseudomonas aeruginosa* lasB‐gfp and rhlA‐gfp	Mature biofilm (16‐h‐old biofilm)	*Cetrelia braunsiana*	Ethyl acetate	QS inhibition assays: 63.8% inhibition of green fluorescent protein (GFP) at 24.7 µg/mL	Quorum sensing inhibition	[34]
	**Endolichenic fungi**					
*Pseudomonas aeruginosa* PAO1	Biofilm formation	*Aspergillus quandricinctus*	Methanol	MBIC = 2.5 mg/mL MBEC > 2.5 mg/mL	Inhibition of QS, of elastase and protease and of exopolysaccharides production	[36]
			Acetone	QS inhibition assays: 80% of violacein production and 77% of proteolytic activity at 6 mg/mL Inhibition of 50% of biofilm formation at 4 mg/mL		
*Pseudomonas aeruginosa* PAO1	Biofilm formation	*Daldinia starbaeckii* (endolichenic fungus)	Water extract + nanoparticles	Inhibition of 82% of biofilm formation at 1.5 µg/mL	Inhibition of bacterial protein synthesis and enzyme production	[37]

Abbreviations: BIC, biofilm inhibitory concentration; MBEC, minimal biofilm eradication concentration; MBIC, minimal biofilm inhibitory concentration; ND, not determined; OD, optical density; QS, quorum sensing.

In conclusion, the available data result from the preparation of extracts produced from seven lichen species that were extracted and tested against a range of bacterial biofilms. Common epiphytic lichens (*P. furfuracea*, *E. prunastri*, *H. tubulosa*, and *H. physodes*) were among these lichen species. One foliaceous lichen (*C. foliacea*) and one crustaceous (*P. muralis*) were tested. Finally, five *Cetrelia* species were studied after their mycobiont culture. The targeted bacterial biofilms were produced by *S. aureus* and *E. faecalis* for Gram+ and *L. pneumophila*, *P. mirabilis*, and *P. aeruginosa* as Gram− species. It is difficult to compare the results of the different studies as the authors did not use the same experimental approaches to produce the biofilm and to assess the inhibition obtained after contact with the extracts. Similarly, the contact times and concentrations used may vary. These factors show just how useful it would be to move toward more standardized tests.

#### 2.1.2 Endolichenic Fungal Extracts

The fungus *Aspergillus quandricinctus* (CBS 135.52) was isolated from the lichen *Usnea longissima*. Its acetone extract was obtained after culture on a PDB medium [36]. Its activity against *P. aeruginosa* biofilm was evaluated through quorum‐sensing inhibition and biofilm formation inhibition. Results showed that the acetone extract significantly inhibited 50% of biofilm formation at 4 mg/mL and also acted as a quorum‐sensing inhibitor at 6 mg/mL without disrupting the bacterial cell growth [36]. The extract also inhibited elastase and protease activities of *P. aeruginosa* as well as exopolysaccharide production involved in the bacterial virulence. The activity was equivalent to that of naringenin used as a reference at 6 mg/mL (60% of biofilm inhibition) (Table 1a). The endolichenic fungus *Daldinia starbaeckii* (DSF), isolated from the lichen *Roccella montagnei*, has been recently studied for its anti‐biofilm activity [38]. An aqueous extract was prepared from a mycelium culture in a PDB medium, and the extract was placed on silver NPs on urinary catheter tubes. The fungal extract was then tested in a surface‐modified catheter model inoculated with *P. aeruginosa* to evaluate its ability to prevent biofilm formation. The experiment showed a dose‐dependent effect with only 18% of biofilm production with a 1.5 µg/mL DSF‐AgNPs catheter. Microscopic analysis showed a reduced exopolysaccharide secretion. Nevertheless, the metabolite composition of DSF is still unstudied by researchers.

The number of endolichenic fungal extracts studied for their anti‐biofilm activity is still limited. The latest research provides interesting anti‐biofilm activities coupling nanoparticle technology with the bioactivity of endolichenic fungi.

### 2.2 Pure Compounds

**FIGURE 2:**
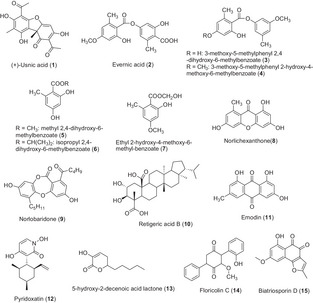
Structures of compounds with activity against bacterial and fungal biofilms produced by lichens and endolichenic fungi.

Several pure compounds (**1**–**9**) (Figure 2) found in lichens and/or their microbiome have been described as having an effect on bacterial biofilms (Table 1b). However, only widespread metabolites have been evaluated for their anti‐biofilm activities (dibenzofurans, depsides, and xanthones).

**TABLE 1b:** Metabolites isolated from lichens and their microbiome and their activity against bacterial biofilms.

Targeted bacterial strain	Targeted biofilm phase	Compound	Quantification of the activity on biofilm	Mechanism of action	Refs.
*Staphylococcus aureus* Seattle 1945 transformed with a GFP plasmid	Biofilm formation	(+)‐Usnic acid (**1**) (loaded in polymers)	Only 1% of attached cells after 24 h of treatment	ND	[42]
*Pseudomonas aeruginosa* pMF230			ND	Mound‐ and mushroom‐shaped microcolonies separated by interstitial void areas Biofilm thicker (scanning confocal laser microscopy) Action on QS	
*Staphylococcus aureus* (Sa3 and Sa15 MRSA)	Adhesion and biofilm formation	Usnic acid (stereochemistry not precise)	Inhibition of 64%–87% of biofilm at 8 µg/mL	Dose‐dependent anti‐adhesive effect	[30]
*Streptococcus* clinical isolates (SP5, SP7, SP9, SP31)	Biofilm formation	(+)‐Usnic acid (**1**)	SP5: Reduction of 60% of biofilm at 10 µg/mL SP9, SP7, and SP31: Reduction of 15%–48% of biofilm at 55 µg/mL	Alteration of proteins, polysaccharides, and fatty acids	[44]
*Pseudomonas aeruginosa* PAO1 (wild type) and rhlA‐gfp, lasB‐gfp (fluorescent strains)	Biofilm formation	Evernic acid (**2**)	Inhibition of 40% of biofilm at 116 µM	Inhibition of QS	[45]
*Legionella pneumophila* Philadelphia 1 strain JCM7571	Biofilm formation Mature biofilm (96‐h‐old biofilm)	3‐Methoxy‐5‐methylphenyl 2,4‐dihydroxy‐6‐methylbenzoate (**3**)	Inhibition of 80% of biofilm formation of at 4 µg/mL MBEC: 37.3 µg/mL	ND	[33]
	Biofilm formation	3‐Methoxy‐5‐methylphenyl 2‐hydroxy‐4‐methoxy‐6‐methylbenzoate (**4**)	Inhibition of 80% of biofilm at 4 µg/mL		
		Methyl 2,4‐dihydroxy‐6‐methylbenzoate (**5**)	Inhibition of 65.1% of biofilm formation at 53.3 µg/mL		
		Isopropyl 2,4‐dihydroxy‐6‐methylbenzoate (**6**)	Inhibition of 54.1% of biofilm formation at 13.3 µg/mL		
		Ethyl 2‐hydroxy‐4‐methoxy‐6‐methyl‐benzoate (**7**)	Inhibition of 68.7% of biofilm formation at 53.3 µg/mL		
*Staphylococcus aureus* 8325–4	Adhesion Biofilm formation	Norlichexanthone (**8**)	Aggregation divided by four at 5 µg/mL Inhibition of biofilm formation 90% at 5 µg/mL	Involvement on regulatory pathway without causing agr dysfunction	[46]
*Pseudomonas aeruginosa*	Biofilm formation Quorum sensing inhibition	Norlobaridone (**9**)	Inhibition of 64.4% of biofilm at 5 µg/mL	Competition with the native LasR agonist (odDHL)	[47]

First, usnic acid, one of the best‐known lichen compounds, produced by several lichen species (*Usnea* sp., *Flavoparmelia caperata*, *Cladonia* sp., *Leprocaulon microscopicum*) [39, 40], showed an inhibitory effect on the biofilm of several bacteria. Only the enantiomer (+)‐usnic acid (**1**) has been evaluated, but sometimes the stereochemistry of the tested compound was not specified. Usnic acid is already known to possess antimicrobial activity against a number of planktonic aerobic and anaerobic bacteria, such as *S. aureus*, *Enterococcus faecium*, *E. faecalis*, *Propionibacterium acnes*, *Bacteroides* sp., and *Clostridium perfringens* [41]. A study conducted by Francolini et al. highlighted the possibility of using (+)‐usnic acid–modified materials for the construction of biofilm‐resistant catheters. Indeed, they demonstrated that (+)‐usnic acid loaded at 2% (w/v) in modified polyurethane, under laminar flow conditions, killed 99% of the attached cells of *S. aureus*, limiting its biofilm formation, and altered the morphology of the *P. aeruginosa* biofilm, suggesting only an impact on the signaling pathways [42]. In addition, Pompilio et al. tested usnic acid and atranorin against methicillin‐resistant *S. aureus*. Their results confirmed the previous ones as usnic acid affected both adhesion and biofilm formation on various polymer surface‐based medicinal implants [43]. Usnic acid inhibited 87% of a 24‐h‐old biofilm at 8 µg/mL, whereas a concentration of 80 µg/mL was needed for atranorin to get the same inhibition activity after 24 h of contact with the compound. Nevertheless, the stereochemistry of usnic acid was not mentioned in the article. Furthermore, (+)‐usnic acid was also evaluated against biofilms of various serotypes of *Streptococcus pyogenes*, a devastating human pathogen responsible for complications of superficial infections [44]. The authors suggested that the anti‐biofilm activity of (+)‐usnic acid varied according to the different serotypes. Depending on serotypes, a concentration ranging between 10 and 70 µg/mL was required to inhibit the biofilm formation. This variable concentration may be due to differences in biofilm composition (e.g., the nature of exopolysaccharides). FT‐IR analysis revealed that usnic acid affected various cellular components present in *S. pyogenes* biofilms. Spectra showed a clear reduction of proteins and polysaccharides on the treated cells, but (+)‐usnic acid may also interfere with the fatty acid components of the biofilm. These results suggested a degradation of amyloid‐like protein fibers and a degradation of EPS layer [44]. Another compound, evernic acid, was also investigated against bacterial biofilms. Evernic acid (**2**) is a polyhydroxylated didepside found in the lichen oakmoss, *E. prunastri*, which possesses anti‐bacterial properties, more specifically against *Legionella* spp. [32]. Derivatives of this didepside, as well as phenol and isochromen derivatives obtained from *E. prunastri*, were evaluated on *L. pneumophila* biofilms by Nomura et al. [33]. Two didepsides (**3** and **4**) inhibited biofilm formation in a dose‐dependent manner, inducing 23% and 18% of inhibition, respectively, at 4 µg/mL. Three phenol derivatives (methyl 2,4‐dihydroxy‐6‐methylbenzoate (**5**); isopropyl 2,4‐dihydroxy‐6‐methylbenzoate (**6**); and ethyl 2‐hydroxy‐4‐methoxy‐6‐methylbenzoate (**7**)) also decreased biofilm formation with 34% of inhibition at 53 µg/mL for phenol **5**, 45% at 13 µg/mL for phenol **6,** and 31% at 53 µg/mL for phenol **7**. Conversely, four other compounds (structures not shown) promoted the biofilm formation at tested concentrations. Gökalsin and Sesal have investigated the usefulness of evernic acid to reduce virulence factors of *P. aeruginosa* by inhibiting QS and biofilm formation [45]. They showed that evernic acid (**2**) at 116 µM decreased the formation of *P. aeruginosa* biofilm by 40%. Measurements with fluorescent strains (rhlA‐gfp and lasB‐gfp) indicated that compound **2** inhibited approximately 50% of the QS system of *P. aeruginosa* at 116 µM. Thus, evernic acid could be promising as a future anti‐QS drug. Norlichexanthone (**8**) is a xanthone commonly found in lichens but also in endophytic fungi. This compound has been found to significantly reduce the formation (90%) and the aggregation (75%) of the *S. aureus* biofilm at 5 µg/mL. The authors found that the biofilm inhibition induced by norlichexanthone was mediated by a regulatory pathway without causing the agr QS system dysfunction [46]. Recently, Soltane et al. studied the QS inhibition activity of norobaridone (**9**), a depsidone isolated from *Parmotrema tinctorum* [47]. An inhibition of 64.6% of the biofilm was observed at a concentration of 5 µg/mL. The agonist and antagonist effects of norlobaridone on LasR, Rh1R, and QscR receptors have been evaluated. The compound shows a selective and competitive inhibition of LasR.

It can be, therefore, noted that only four lichenic compounds, belonging to four different classes (dibenzofurans, depsides, depsidones, and xanthones), have already been evaluated for their anti‐biofilm effects.

## 3 Extracts and Compounds With Activity Against Fungal Biofilms

The fungal pathogen most commonly associated with biofilm infections is *C. albicans*, the resulting infections of which can be linked to a high morbidity and mortality. Other biofilm‐forming *Candida* species include *Candida parapsilosis*, *Candida tropicalis*, *Candida krusei*, and *Candida glabrata*, which is also responsible for serious infections. Other fungal species, such as *Cryptococcus neoformans*, *Coccidioides immitis*, *Aspergillus* spp., *Fusarium* spp., *Blastoschizomyces capitatus*, *Malassezia pachydermatis*, *Pneumocystis* spp., *Trichosporon asahii*, *Rhizopus* spp., and *Rhizomucor* spp., are also described as causative agents of biofilm‐related fungal infections [48]. They are often associated with a high level of resistance requiring the discovery of new active molecules. As for the study of bacterial biofilms, several in vitro methods are used by scientists to assess the susceptibility of biofilms, such as MBIC and MBEC. The most common assays are made with a static model, and the biofilm is quantified based on the metabolic activity (XTT or MTT tests) or on their biomass (crystal violet test) [49]. To determine their ability to inhibit the biofilm formation, compounds or extracts are incorporated during the biofilm growth phase, either before the adhesion phase, at the very beginning of the process, or a little later, just after the yeasts have adhered. The MBIC is the minimum concentration resulting in 100% inhibition of the biofilm formation after 24 or 48 h of contact with the compounds or the extract.

### 3.1 Extracts

Few studies have been carried out on fungal biofilms assessing lichen or lichenic microbiome extracts (Table 2a). Recently, a screening of 38 acetonic lichen extracts revealed that the incubation for 24 or 48 h of pre‐adhered *C. albicans* yeasts with a lichen extract prepared from *Cladonia ramulosa*, *Cladonia uncialis*, *E. prunastri*, *Peltigera hymenina*, *Ramalina fastigiata*, and *Xanthoparmelia conspersa* species significantly inhibited the biofilm maturation phase (IC_50_ < 50 µg/mL). Interestingly, these six lichens displayed both anti‐maturation and anti‐biofilm activities (24‐h‐old biofilm treated for 24 and 48 h) (half‐maximal inhibitory concentrations IC_50__mat and IC_50__biof ≤100 µg/mL after 48 h of treatment). The two common epiphyte lichens in temperate European countries, *E. prunastri* and *R. fastigiata*, were the most active with IC_50__mat <4 µg/mL and IC_50__biof <10 µg/mL after 48 h of contact. On the basis of bibliographic data and chromatographic analyses, evernic acid was suspected to be responsible for the activity. Additionally, microscopic observations performed using trypan blue staining showed that yeasts growing as biofilm were still alive after the treatment, suggesting that active extracts may act thanks to a dispersant or removing action that would not disrupt the fungal cell membrane. This screening, restricted to lichens located in the center of France, described the anti‐biofilm activity of 38 acetonic extracts. This preliminary step has given some elements to further research some specific metabolites.

**TABLE 2a:** Lichen and lichenic microbiome extracts and their activity against fungal biofilms.

Targeted fungal strain	Targeted biofilm phase	Source	Type of extract	Quantification of the activity on biofilm	Mechanism of action	Refs.
*Candida albicans* ATCC 3153	Biofilm Maturation (2 h adherent cells treated during 24 or 48 h) Mature biofilm (24‐h‐old biofilm treated 24 or 48 h)	*Cladonia ramulosa*	Acetone	IC_50__mat 50 µg/mL and IC_50__biof 50 µg/mL after 48 h	Probably dispersant or removing action that would not disrupt the fungal cell membrane	[54]
		*Cladonia uncialis*		IC_50__mat 25 µg/mL and IC_50__biof <10 µg/mL after 48 h		
		*Evernia prunastri*		IC_50__mat = 1.56 µg/mL and IC_50__biof <10 µg/mL after 48 h		
		*Peltigera hymenina*		IC_50__mat = 12.5 µg/mL and IC_50__biof 50 µg/mL after 48 h		
		*Ramalina fastigiata*		IC_50__mat = 3.125 µg/mL and IC_50__biof <10 µg/mL after 48 h		
		*Xanthoparmelia conspersa*		IC_50__mat 25 µg/mL and IC_50__biof <10 µg/mL after 48 h		
		*Xanthoparmelia tinctina*		IC_50__mat 6.25 µg/mL and IC_50__biof 100 µg/mL after 48 h		
*Candida albicans* ATCC 3153	Biofilm maturation (2 h adherent cells treated during 48 h) Mature biofilm (24‐h‐old biofilm treated during 48 h)	*Anthostomella pinea*	Ethyl acetate obtained after culture on PDA medium, MEA medium, and Sabouraud medium	Biofilm maturation: 65%–71% of inhibition at 100 µg/mL Mature biofilm: 50%–70% of inhibition at 100 µg/mL	ND	[55]
	Biofilm maturation (2 h adherent cells treated during 48 h) Mature biofilm (24 h old biofilm treated during 48 h)	*Preussia persica*	Ethyl acetate obtained after culture on PDA medium, MEA medium, and Sabouraud medium	Biofilm maturation: 50%–64% of inhibition at 100 µg/mL Mature biofilm: 68% of inhibition at 100 µg/mL		

### 3.2 Pure Compounds

#### 3.2.1 Isolated From Lichens

Usnic and evernic acids (**1** and **2**), previously mentioned as active against bacterial biofilms, were also evaluated against fungal biofilms (Table 2b). (+)‐Usnic acid was tested both against planktonic and sessile cells of *C. albicans*. The dibenzofuran‐like compound showed no antifungal effect compared to fluconazole even with a concentration of 100 µg/mL [50]. But, at 100 µg/mL, (+)‐usnic acid reduced the biofilm formation by 65% and reduced the cell viability of the mature biofilm. At this concentration, it inhibited the yeast to hyphal switch, reduced aggregation of cells, and reduced the thickness of the matured biofilms. The authors noted a decrease in the hydrophobicity of *Candida* cells in the presence of (+)‐usnic acid, which was able to reduce various sugars of the exopolysaccharide layer. Another study showed that (+)‐usnic acid used at 4 µg/mL against mature biofilms inhibited by 71% the biofilms produced by azole‐resistant strains and by 87% those produced by azole‐sensitive strains [51]. In this study, authors also evaluated the anti‐biofilm activity of usnic acid against *Candida orthopsilosis* and *C. parapsilosis* strains [52]. Overall, their results showed that the anti‐biofilm activity of usnic acid (reduction of metabolic activity of biofilm [BEC_80_] of 31.2 and 62.5 µg/mL, respectively) was weaker than its antifungal activity, regardless of the susceptibility profile of tested strains (planktonic growth: IC_80_ 7.8 and 15.6 µg/mL, respectively). Moreover, *C. orthopsilosis* was more susceptible to usnic acid than *C. parapsilosis*, with a minimum biofilm fungicidal concentration (MBFC) of 125 µg/mL (vs. 250 µg/mL for *C. parapsilosis*). The lichen depside, evernic acid (**2**), showed both anti‐maturation (minimal maturation inhibition concentration MMIC_50_ = 6.25 µg/mL after 48 h of contact) and anti‐biofilm (minimal biofilm inhibitory concentration MBIC_50_ = 12.5 µg/mL after 48 h of contact) activities against *C. albicans* [53]. Scanning electron microscopy observations performed after treatment with this compound highlighted some wrinkled yeasts, and a matrix that was more condensed with a lesser coating aspect, suggesting an effect on yeast cell morphology and a modification of the extracellular matrix distribution. The authors extended the tests to other lichenic compounds, two other depsides, squamatic and thamnolic acids. These showed only a significant anti‐maturation effect with MMIC_50_ = 12.5 µg/mL after 48 h of contact. Atranorin and gyrophoric acid displayed no activity. In the same study, some depsidones (norstictic acid, salazinic acid, stictic acid, physodic acid, and 3‐hydroxyphysodic acid) were also investigated without any success. Retigeric acid B (RAB) (**10**) is a lichen‐derived pentacyclic triterpenoid isolated from *Lobaria kurokawae*. Chang et al. showed that this compound displayed synergistic antifungal activity against *C. albicans* in the presence of azoles. These combinations also acted synergistically to block the formation of biofilm regardless of azole‐sensitive or azole‐resistant strains. Indeed, the biofilm formation of four strains of *C. albicans* was reduced by 80% using RAB at 8 µg/mL combined with fluconazole [0.125–0.5] µg/mL versus RAB >32 µg/mL alone or fluconazole >2 µg/mL alone. The same observation was done with ketoconazole and itraconazole in combination with RAB. This effect could be explained by the observed attenuation of yeast‐to‐hyphal switch [57]. Scanning confocal laser microscopy observations showed that when exposed to a RAB–fluconazole combination, no biofilm was observed; only clusters of yeast cells adhered to the substratum were present. qPCR analyses revealed a reduction of the transcriptional expression of MDR1, which is a multidrug efflux pump implicated in biofilm resistance. Emodin (**11**) is a widely spread anthraquinone in the plant and fungal kingdoms [58]. This metabolite is found in lichens as well as in filamentous fungi and has been shown to display interesting properties against planktonic and biofilm *C. albicans* yeasts. Indeed, this compound demonstrated antifungal activity against several *Candida* species (*C. albicans*, *C. krusei*, *C. parapsilosis*, and *C. tropicalis*) with an MIC value of 12.5–50 µg/mL. This activity was confirmed on clinical strains of *Candida* and was significantly higher than that against yeast living in biofilms. The authors worked on 50 clinical strains and showed that the compound inhibited adhesion in 30 of them, and mature biofilm in only 15. The anti‐biofilm effect could be due to the observed inhibition of hyphal formation and growth, as fluorescent images revealed that 6 µg/mL of emodin totally inhibited hyphal formation. This compound also inhibited protein kinases by 50% at concentrations starting from 0.5 µg/mL. It inhibited the phosphorylation of many cellular proteins, presumably due to the inhibition of protein kinase CK2.

**TABLE 2b:** Metabolites isolated from lichens and lichens microbiome (including fungal cultures) and their activity against fungal biofilms.

Targeted fungal strain	Targeted biofilm phase	Compound	Quantification of the activity on biofilm	Mechanism of action	Refs.
*Candida albicans* ATCC 90028	Biofilm formation Mature biofilm (48‐h‐old biofilm treated 5 h)	(+)‐Usnic acid (**1**)	Inhibition of 65% of biofilm at 100 µg/mL	Reduction of metabolic activity, aggregation, and hydrophobicity of *Candida* cells. Prevention of hyphal formation (scanning confocal laser microscopy) and reduction of various sugars of EPS layer	[50]
Azole‐resistant *Candida albicans* strain RCa	Mature biofilm (48‐h‐old biofilm treated 48 h)	(+)‐Usnic acid (**1**)	Inhibition of 71% of biofilm at 4 µg/mL	Alteration of the prooxidant‐antioxidant balance Reduction of the biofilm mass and thickness (scanning confocal laser microscopy)	[51]
Azole‐sensitive *Candida albicans* strain SCa			Inhibition of 88% of biofilm at 4 µg/mL		
*Candida orthopsilosis* isolate	Mature biofilm (24‐h‐old biofilm treated 48 h)	Usnic acid (stereochemistry not precise)	BEC80: 31.2 µg/mL MBFC 125 µg/mL	ND	[56]
*Candida parapsilosis* isolate			BEC80: 62.5 µg/mL MBFC 250 µg/mL		
*Candida albicans* ATCC 28367	Maturation phase (2‐h‐old biofilm treated 24–48 h) Mature biofilm (24‐h‐old biofilm treated 24–48 h)	Evernic acid (**2**)	MMIC_50_ = 6.25 µg/mL after 48 h of contact MBIC_50_ = 12.5 µg/mL after 48 h of contact	Effect on yeast cell morphology and modification of the extracellular matrix distribution (SEM observation)	[53]
*Candida albicans* isolates YEM30, SC5314, CA2, CA10, and CASA1 with GFP‐tagged CDR1	Biofilm formation (48 h treatment)	Retigeric acid B (**10**)	Inhibition of 80% of biofilm with 8 µg/mL RAB and [0.125–0.5] µg/mL fluconazole or [0.008–0.032] µg/mL ketoconazole or [0.008–0.064] µg/mL itraconazole in combination	Attenuation of yeast‐to‐hyphal switch (scanning confocal laser microscopy) Fungicidal activity Reduction of the transcriptional expression of MDR1	[57]
*Candida albicans* ATCC 10231*, Candida parapsilosis* ATCC 22019, *Candida krusei* ATCC 14243, and *Candida tropicalis* ATCC 13803 + 15 clinical strains of *Candida albicans* isolated from gynecological patients	Adherence phase (treatment of 1.5 h) Mature biofilm (48‐h‐old biofilm treated 24 h)	Emodin (**11**)	Suppression of adhesion of 30/50 clinical strains Significant anti‐biofilm effect on 15/50 clinical strains	Inhibition of hyphal formation and growth (light and fluorescence microscopy) Protein kinase CK2 inhibition	[58]
*Candida albicans* YEM 30	Biofilm formation (48 h treatment)	Pyridoxatin (**12**)	Inhibition of 70% with 4 µg/mL	No effect on transition yeast to hyphae Reduction of expression of genes involved in ergosterol biosynthesis causing arrested growth Decrease of ergosterol and increase of lanosterol and squalene	[59]
Clinical isolates: *Candida albicans* (*n* = 15) *Candida glabrata* (*n* = 26) *Candida krusei* (*n* = 17) *C. parapsilosis* (*n* = 8) *C. tropicalis* (*n* = 10) *C. orthopsilosis* (*n* = 8) *Candida rugosa* (*n* = 5) *Candida dubliniensis* (*n* = 1) *Meyerozyma guilliermondii* (*n* = 1) *Candida metapsilosis* (*n* = 1)	Mature biofilm (24‐h‐old biofilm treated 48 h)	5‐Hydroxy‐2‐decenoic acid lactone (HDAL) (**13**)	MBIC_50_ between 128 and 512 µg/mL	ND	[61]
*Candida albicans* SC5314 and TDH3‐GFP‐CAI4	Mature biofilm (48‐h‐old biofilm treated 24 h)	Floricolin C (**14**)	Reduction of 58% of the biofilm at 8 µg/mL	Fungicidal effect Little effect on the inhibition of yeast‐to‐hyphae transition	[63]
*Candida albicans* SC5314	Adherence phase (incubation of 24 h of *Candida* cells with the drug)	Biatriosporin D (**15**)	Reduction of 70% of biofilm at 8 µg/mL	Inhibition of hyphal morphogenesis	[64]

Abbreviations: BEC, biofilm reduction metabolic activity concentration; MBFC, minimum biofilm fungicidal concentration; MBIC, minimum biofilm inhibitory concentration; MMIC, minimal maturation inhibition concentration; ND, not determined.

Therefore, it can be noted that only four lichenic compounds, belonging to four different classes (dibenzofurans, depsides, triterpenoids, and anthraquinone), have already been evaluated for their anti‐biofilm effects against *Candida* biofilms.

#### 3.2.2 Isolated From Endolichenic Fungi

Pyridoxatin (**12**) is a compound isolated from *Tolypocladium cylindrosporum*, an endolichenic fungus derived from the lichen *Lethariella zahlbruckneri*. Its effect on the formation of *C. albicans* biofilm was evaluated by growing biofilm for 48 h in the presence of this compound. The concentration of 4 µg/mL led to the reduction of biofilm formation by 70% compared with the control [59]. This N‐containing compound (Figure 2) also displayed antifungal activity against several *Candida* species growing planktonically (*C. albicans*, *C. krusei*, *C. glabrata*, and *C. tropicalis*). Additional experiments revealed no effect of this compound on the *C. albicans* yeast‐to‐hyphal transition, a critical virulence factor. Conversely, based on qPCR analyses, it was shown to reduce the expression of genes implicated in ergosterol biosynthesis, and LC–MS analyses showed a decrease of ergosterol and an increase of lanosterol and squalene, providing the first clues to understanding its mechanism of action. Bioguided fractionation of an *Aureobasidium pullulans* extract was conducted in order to isolate 5‐hydroxy‐2‐decenoic acid lactone (HDAL) (**13**). This fungus is frequently observed as an inhabitant of several lichen species (*Lobaria*, e.g.) and is also found in soil [60]. The compound HDAL has been evaluated against *C. albicans* 24‐h‐old biofilms and against biofilms developed by other *Candida* species as well. The average MIC required to inhibit by 50% the biofilm (MIC_50_) was 256 µg/mL against *C. albicans* isolates, whereas *C. parapsilosis*, *C. krusei*, *C. orthopsilosis*, *Meyerozyma guilliermondii*, and *Candida metapsilosis* isolates were more sensitive with an MIC_50_ value of 128 µg/mL [61]. The authors highlighted that HDAL demonstrated a large spectrum of inhibitory action against biofilms of several *Candida* species, provided very high concentrations (at least 128 µg/mL) were used. Another quinonoid compound, isolated from the endolichenic fungus *Floricola striata* in *Umbilicaria* sp. thallus, was identified as an anti‐biofilm compound toward *C. albicans* [62]. Results demonstrated that floricolin C (**14**) reduced biofilm formation in a dose‐dependent manner, and a reduction by 50% was observed with a concentration of 8 µg/mL. Furthermore, this inhibitory activity was also observed against preformed mature biofilms (48 h), with more than 60% of the biofilm being killed when treated with 32 µg/mL of floricolin C (XTT reduction assay). This compound exerts a simultaneous fungicidal effect [63]. Biatriosporin D (**15**) was isolated from the endolichenic fungus *Biatriospora* sp. This compound also exerts a dose‐dependent anti‐biofilm activity against *C. albicans* with a concentration of 4 µg/mL [64]. At 8 µg/mL, the biofilm was completely destroyed by biatriosporin D. The observed effect (in vitro XTT assay and microscopic observation) was due to the inhibition of *C. albicans* hyphal morphogenesis. So, four metabolites issued from the culture of endolichenic fungi have been tested for their anti‐biofilm activity against *Candida* biofilm. These metabolites belong to four different chemical classes.

## 4 Conclusion

This study highlights the fact that lichens and their associated microbial metabolites are still poorly explored for their biological properties, especially regarding their anti‐biofilm activity. This can be explained by the fact that lichens and their microbiomes have been studied less than plants. Fortunately, over the past few years, the number of fungi and bacteria isolated from lichens has increased, and original molecules have been isolated and characterized [8, 65]. Extracts are still more studied for their biological activity than pure compounds. Their exact chemical composition is sometimes partially known and described, which is damaging.

Moreover, fungal biofilms are still less studied than bacterial biofilms regarding the number of tested compounds. Except for usnic acid and evernic acid, which are the most studied lichen metabolites, compounds are rarely evaluated against both fungal and bacterial biofilms. Among the strains used, *S. aureus*, *P. aeruginosa*, and *C. albicans* are the most studied.

The methods for evaluation of anti‐biofilm activity vary. Several phases are targeted: adhesion, formation, and activity against a mature biofilm, the second one being the most studied. For fungal strains, cell viability is analyzed using MTT (3‐(4,5‐dimethylthiazol‐2‐yl)‐2,5‐diphenyl tetrazolium bromide) or XTT. The optical density measurement or microscopic observations are also used in some studies, whereas qPCR analyses can complete some studies. For bacterial strains, the crystal violet assay is mostly used for anti‐biofilm tests. The standardization of protocols for the evaluation of anti‐biofilm activity could be helpful in order to compare the activity of each compound and extract. Some hypotheses of the mechanism of action are sometimes proposed. For bacterial biofilm, QS inhibition is often mentioned with effect on the production of proteins, polysaccharides, and fatty acids in the biofilm. For fungal biofilm, the yeast‐to‐hyphal transition is often implicated with effects on ergosterol synthesis or modification of cell morphology or matrix composition.

The results obtained for extracts on bacterial biofilm could give rise to further investigation of the following pure lichenic compounds: caperatic, atraric, protocetraric, physodic, and olivetoric acids, as well as atranorin and chloroatranorin, which have been identified as the main compounds of the extracts. In the same way, considering the activity observed in fungal biofilms for the extracts, it could be interesting to further evaluate the activity of other lichen metabolites.

## Conflicts of Interest

The authors declare no conflicts of interest.

## Data Availability

All data analysed during this study are included in this published article.
